# Structural Homology Fails to Predict Secretion Efficiency in *Pichia pastoris*: Divergent Responses of Architecturally Similar scFvs to Multi-Parametric Genetic Engineering

**DOI:** 10.3390/ijms26104922

**Published:** 2025-05-21

**Authors:** Ningning Wang, Yang Xiao, Xiyu Liu, Yuanqing Li, Dehua Yu, Jia Guo, Ping Lu, Xiaopeng Zhang

**Affiliations:** 1 Institute of Physical Science and Information Technology, Anhui University, Hefei 230601, China; 13461985923@163.com; 2 Laboratory of Advanced Biotechnology, Beijing Institute of Biotechnology, Beijing 100071, China; lxy9481_amms@126.com; 3 Nanhu Laboratory, Jiaxing 314051, China; xy@nanhulab.ac.cn (Y.X.); lllllliyuanqing@163.com (Y.L.); m15968852573@163.com (D.Y.); 13252363518@163.com (J.G.); pingluu0127@163.com (P.L.)

**Keywords:** *Pichia pastoris*, scFv, secretory pathway engineering, gene dosage optimization, expression cassette design

## Abstract

AI-driven biologics manufacturing demands an efficient protein production platform. In this study, we optimized scFv secretion in *Pichia pastoris* through three strategies: gene dosage optimization, expression cassette design, and endoplasmic reticulum (ER) secretory pathway reprogramming. Using two structurally homologous scFv variants—PR961 and PR953—with divergent basal secretion levels (12.35:1 ratio), we demonstrate that protein-specific thresholds govern optimization efficacy. While increasing gene copy numbers yielded limited improvements (PR961: 1.25-fold at four copies; PR953: 2.37-fold at six copies), reconfiguring the expression cassette to a V_H_-linker-V_L_ orientation significantly enhanced secretion (11.18-fold for PR961; 5.09-fold for PR953). Twenty-one genes in three functional modules of the secretory pathway were knocked out or overexpressed. The pathway reprogramming results revealed distinct regulatory dependencies: PR961 secretion relied on ER-to-Golgi trafficking (*SEC23* overexpression: 1.20-fold), whereas PR953 depended more on upstream translocation (*SEC62*: 1.66-fold) and oxidative folding (*ERO1*: 1.81-fold) enhancements. Notably, both variants exhibited a glycosylation-dependent regulation through *CNE1*. Our findings challenge the assumption that structural homology (63% amino acid identity; RMSD 0.47 Å) ensures consistent optimization outcomes, highlighting the imperative for protein-tailored engineering strategies in synthetic biology.

## 1. Introduction

Protein engineering is a powerful biotechnology for creating new proteins with specific functions and improved properties [1,2]. Recent advancements in artificial intelligence algorithms, such as AlphaFold, RoseTTAFold, and MMseqs2, have revolutionized de novo protein design, allowing for the rapid generation of structurally refined protein variants [3]. However, due to limitations in AI accuracy, there are often many variants that need to be expressed and screened to obtain the desired protein [4,5]. These AI-designed variants typically share significant sequence and structural similarities [6]. Therefore, a reliable expression platform is crucial for validating AI-designed proteins quickly and efficiently, as well as for facilitating further research and the large-scale production of target proteins.

The methylotrophic yeast *Pichia pastoris* (*P. pastoris*) is a leading microbial host for recombinant protein production in academic and industrial settings. While it is known for successfully producing over 5000 heterologous proteins [7,8,9], its secretion efficiency varies greatly, even among similar proteins. For example, the expression levels of hydrolases with similar three-dimensional structures and functions can differ by nearly 100-fold [10]. This variability may be due to the multiple quality control mechanisms of the host, such as codon usage biases affecting ribosomal elongation rates [11] and the ER retention of misfolded proteins [12]. Unlike *E. coli*, *P. pastoris* employs eukaryotic folding chaperones and vesicular trafficking, enabling it to process complex proteins, but these pathways also require optimization for the expression of specific proteins [13,14].

Various genetic engineering strategies have been developed to optimize the *P. pastoris* expression system for heterogenous protein production. These strategies include increasing the copy number of target genes [15,16], refining transcriptional regulatory elements, and optimizing sequence rearrangement and codon usage [17,18,19,20]. In addition to these traditional methods, the emergence of synthetic biology has introduced new tools and strategies. One such approach involves utilizing CRISPR gene editing tools to modulate key processes in the ER secretory pathway to enhance protein expression efficiency [21,22,23]. The secretory pathway begins with the translocation of nascent peptides into the ER, where they undergo processes of folding, post-translational modification, and quality control, before being transported through vesicles to the Golgi apparatus [24]. Studies have shown that overexpressing folding factors and molecular chaperones, such as protein disulfide isomerase (Pdi) and luminal binding protein (Bip), can improve the folding and secretion efficiency of recombinant proteins in *P. pastoris* [25]. The optimization of protein translocation and the unfolded protein reaction (UPR) has also been demonstrated to significantly enhance the secretion of the human hyaluronidase PH-20 protein in *P. pastoris* [26]. Furthermore, a recent study has indicated that modulating the dynamic regulation of cytosolic and ER Hsp70 cycles, a key regulator of the protein translocation process, can increase protein secretion by up to 5-fold across various protein classes, including antibody fragments [27].

In this study, we investigated three engineering strategies to optimize heterologous protein secretion in *P. pastoris*, using structurally homologous single-chain variable fragment (scFv) variants (PR961 and PR953), with a 12.35-fold difference in baseline expression. By comparing gene dosage optimization, V_H_-V_L_ cassette architecture engineering, and CRISPR-mediated ER secretory pathway reprogramming, we found that structural homology (63% amino acid identity; RMSD 0.47 Å) does not accurately predict optimization results. These results indicate that yeast secretory efficiency is influenced by hidden structural factors that interact with host cell machinery.

## 2. Results

### 2.1. Comparative Analysis of PR961 and PR953 scFv Variants

**Figure 1:**
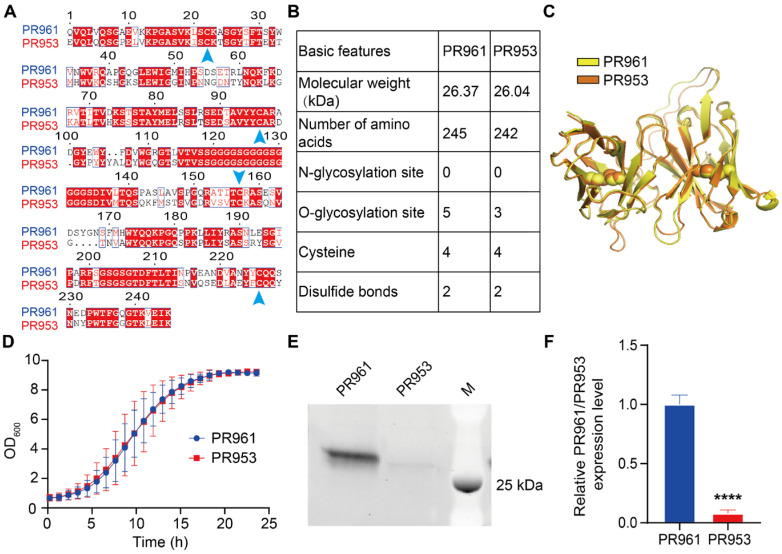
Characteristic comparison of PR961 and PR953. (**A**) Amino acid sequences of the two scFvs were compared using ESPript 3.0. Red boxes highlight homologous regions, and arrows indicate the conserved cysteine positions. (**B**) Basic information for PR961 and PR953, including potential N- and O-glycosylation sites, was predicted using the DTU Health Tech website. (**C**) Overlay of protein crystal structures of PR961 (yellow, PDB ID: 7DET) and PR953 (orange, PDB ID: 7DEU) obtained from RCSB PDB. (**D**) Growth curves of PR961 and PR953-harboring yeast strains incubated for 24 h in MGY medium. (**E**) SDS-PAGE analysis of PR961 and PR953 (V_H_-linker-V_L_, 2 copies) secretion in *P. pastoris*. (**F**) PR961 and PR953 secretion levels were quantified in three independent experiments using ImageJ 1.53a gel analysis software. Statistical significance is denoted as *p* < 0.0001 (****).

Two SARS-CoV-2 scFv variants [28], PR961 and PR953, were subjected to comprehensive sequence–structure–function analysis. The analysis using the ESPript 3.0 demonstrated a 63.16% amino acid sequence identity and comparable molecular weights between the two scFvs: PR961, 26.37 kDa (245 aa); PR953, 26.04 kDa (242 aa) (Figure 1A,B). Predictions by DTU Health Tech tools (https://services.healthtech.dtu.dk/, accessed on 20 March 2024) indicated five potential O-glycosylation sites for PR961 and three for PR953, with no identified N-glycosylation sites. The protein structures of PR961 and PR953 were obtained from the PDB database and compared using PyMOL 2.5.5 software. The structural superimposition of these two scFvs with PyMOL revealed a conserved structural architecture (RMSD = 0.47 Å) with identical spatial configurations of critical disulfide bonds (Figure 1C). Growth curve tests demonstrated similar growth kinetics for yeast strains carrying PR961 and PR953 with a V_H_-linker-V_L_ architecture (Figure 1D), indicating that scFvs were not toxic to yeast growth. The SDS-PAGE analysis showed a significantly higher secretion of PR961 than PR953 in *P. pastoris* (12.35-fold, *p* < 0.0001, Figure 1E,F). In contrast, both mRNA levels and total intracellular scFv protein expression were comparable between the PR961 and PR953 strains, with no statistically significant differences noted (Figure S1). Based on these characteristics, these two scFvs were selected to further investigate the factors influencing scFv secretion in yeast.

### 2.2. The Limited Effect of Gene Dosage Optimization on Enhancement of PR961 and PR953 Secretion

**Figure 2:**
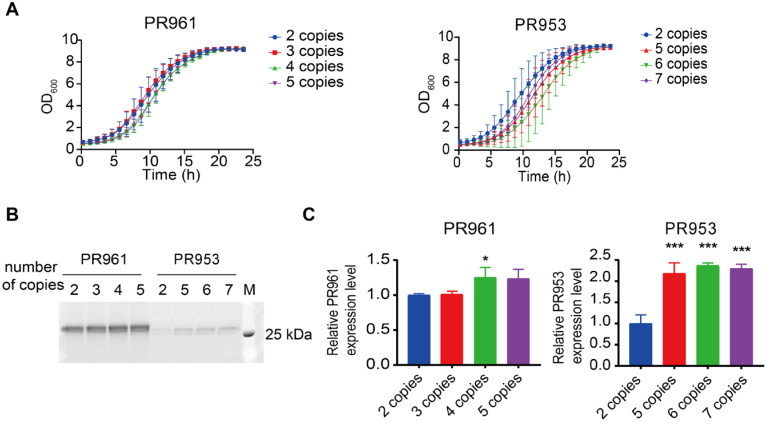
Secretion of PR961 and PR953 in their respective recombinant strains with varying gene dosages. (**A**) Growth curves of PR961 and PR953 strains with different copy numbers incubated for 24 h in MGY medium. (**B**) SDS-PAGE analysis of PR961 and PR953 with different copy numbers. (**C**) PR961 and PR953 secretion levels were quantified in three independent experiments using ImageJ 1.53a gel analysis software. Statistical significance levels are denoted as follows: *p* < 0.05 (*) and *p* < 0.001 (***).

A strong positive correlation between the gene copy number and intracellular protein expression levels has been clearly demonstrated [29,30,31]. Based on this, we evaluated the impact of gene dosage optimization on the secretory expression levels of the two similar scFvs in *P. pastoris*. We first constructed recombinant yeast strains carrying varying copy numbers of scFvs with the V_H_-linker-V_L_ architecture: strains containing two, three, four, and five copies of PR961, as well as strains with two, five, six, and seven copies of PR953 (Figure S2). The analysis of the 24 h growth curves showed that increasing the gene copy number did not significantly affect the growth kinetics of strains, indicating that changes in the gene copy number had a limited impact on the overall growth of the yeast strains (Figure 2A). The SDS-PAGE results showed that the secretion of PR961 increased with copy number from two to four, plateauing at five copies. Similarly, the secretion of PR953 increased from two to six copies, stabilizing at seven copies (Figure 2B). The quantitative analysis showed a maximum 1.25-fold increase in PR961 (four copies) and a 2.37-fold increase in PR953 (six copies) compared to their parental strains (Figure 2C). Notably, even after optimization, the PR953 secretion level remained lower than that of PR961. These findings suggest a maximum copy number limit that regulates the secretion efficiency of scFvs.

### 2.3. V_H_-Linker-V_L_ Construction Enhanced scFv Secretion Compared to V_L_-Linker-V_H_

**Figure 3:**
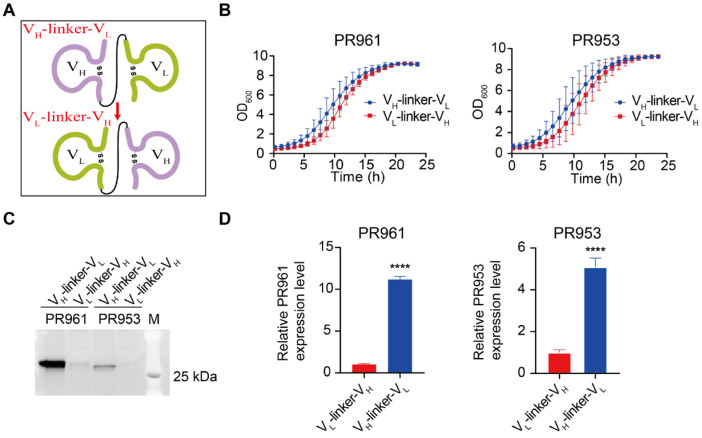
Secretion of PR961 and PR953 in their respective recombinant strains carrying constructs with both V_H_-linker-V_L_ and V_L_-linker-V_H_ orientations. (**A**) Schematic representation of V_H_-linker-V_L_ and V_L_-linker-V_H_ construction orientations for both PR961 and PR953. (**B**) Growth curves of PR961 and PR953 strains transformed with V_H_-linker-V_L_ and V_L_-linker-V_H_ constructs incubated for 24 h in MGY medium. (**C**) SDS-PAGE analysis of PR961 and PR953 in their respective expression strains, carrying constructs with different scFv domain orientations. (D) Secretion levels of PR961 and PR953 were quantified using ImageJ 1.53a software, and statistical significance was determined based on three independent experiments. Statistical significance is denoted as *p* < 0.0001 (****).

An scFv is a type of recombinant antibody that consists of a single polypeptide containing the variable light chain (V_L_) and variable heavy chain (V_H_) of an antibody [32]. To assess the impact of different expression cassette designs on scFv secretion in *P. pastoris*, we constructed strains carrying PR961 and PR953 scFv variants in V_H_-linker-V_L_ or V_L_-linker-V_H_ orientations, each with two-copy genomic integrations (Figure 3A, Figures S2 and S3). The construction orientation showed no growth phenotype alterations (Figure 3B). Moreover, the Octet kinetics analysis revealed that all tested scFv variants, regardless of their domain orientation, retained the capacity to specifically bind to the SARS-CoV-2 RBD (Figure S4 and Table S1). However, the SDS-PAGE analysis revealed significant orientation-dependent secretion differences. The V_H_-linker-V_L_ configuration demonstrated an enhanced secretory capacity, with 11.18-fold (*p* < 0.0001) and 5.09-fold (*p* < 0.0001) improvements for PR961 and PR953, respectively, compared to V_L_-linker-V_H_ counterparts (Figure 3C,D). Compared to gene dosage optimization, this expression cassette design strategy showed superior efficacy in enhancing scFv secretion. Consequently, the V_H_-linker-V_L_ construct was selected for all subsequent experiments in this study.

### 2.4. Disrupting Key ER Components Downregulated scFv Secretion

**Figure 4:**
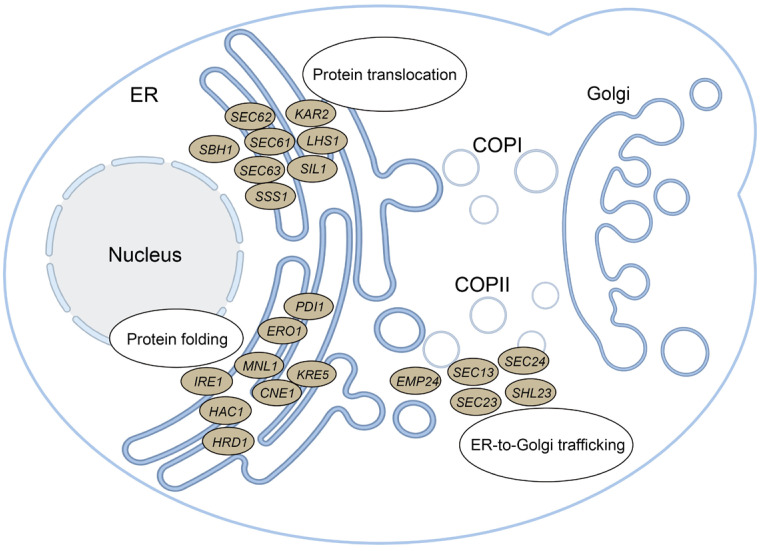
Schematic illustration of the three ER functional modules and the twenty-one specific genes targeted within these modules in this study. The illustration was generated using the BioRender mapping website (https://www.biorender.com/, accessed on 11 March 2025).

Secretory pathway engineering is a widely utilized strategy to enhance recombinant protein production in yeast systems [33]. As the starting point for secretory trafficking, the ER oversees critical processes including protein folding, quality control, and vesicular transport [2]. We selected twenty-one target genes related to three ER functional modules (protein translocation, protein folding, and ER-to-Golgi trafficking), to systematically investigate their impact on scFv expression in *P. pastoris*. The specific genes targeted within the three modules are depicted in Figure 4, and detailed gene information is provided in Supplementary Materials, Table S2.

**Figure 5:**
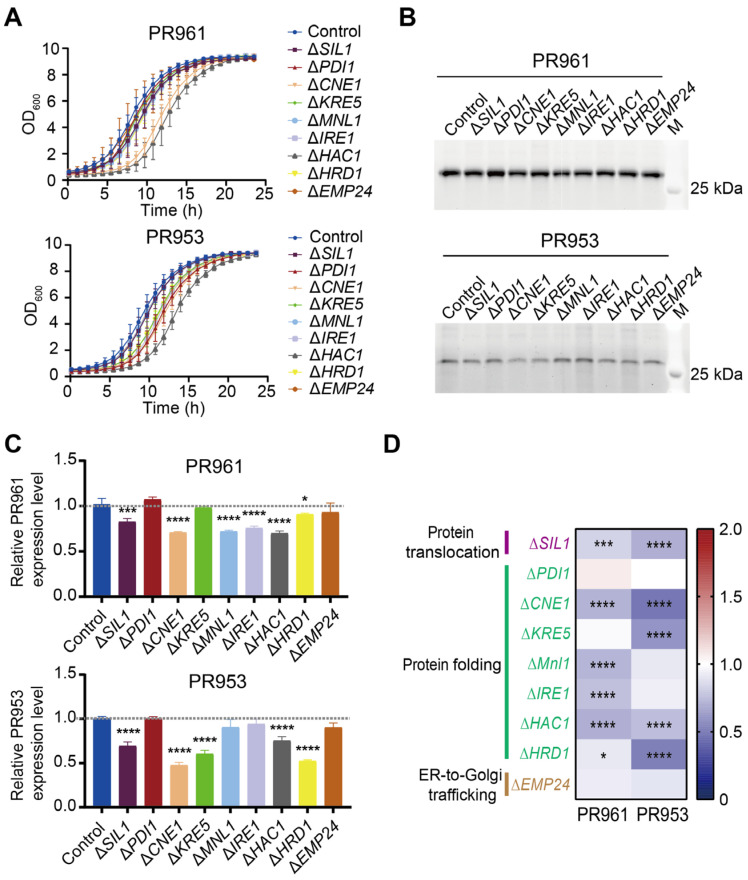
Effects on PR961 and PR953 secretion by disrupting ER-related genes. (**A**) Growth curves of nine distinct single-gene knockout strains of PR961 and PR953 incubated for 24 h in MGY medium. (**B**) SDS-PAGE analysis of PR961 and PR953 secretion levels. The gels were then stained with SYPRO dye. (**C**) Quantification of PR961 and PR953 secretion using ImageJ 1.53a software. Significant differences were determined from three independent experiments. (**D**) Heat map of the fold changes in PR961 and PR953 secretion relative to their respective controls. Statistical significance levels are denoted as follows: *p* < 0.05 (*), *p* < 0.001 (***), and *p* < 0.0001 (****).

Knockout experiments revealed the essentiality of twelve genes for *P. pastoris* cell survival, as detailed in Table S2. These twelve genes include two essential genes (*LHS1* and *SHL23*) identified in the present study and ten essential genes previously reported [13,34,35,36,37]. Consequently, knockout mutant strains for nine of the twenty-one target genes were successfully obtained in our research and were utilized in the subsequent experiments. The growth phenotype analysis showed that the deletion of *CNE1* and *HAC1* significantly delayed yeast cell growth but did not affect the final biomass of both strains at 24 h (Figure 5A and Figure S5A). None of the individual knockout strains targeting these nine genes showed enhanced secretion levels of the two scFvs (Figure 5B,C). These data indicated that disrupting two out of these three ER modules led to a decrease in scFv secretion, except for the ER-to-Golgi trafficking module (Δ*EMP24*, Figure 5D). Notably, the key genes associated with these modules differed between PR961 and PR953 strains. Specifically, disrupting *CNE1* had the most significant inhibitory effect on PR953 secretion (0.48-fold vs. parental control), while PR961 exhibited maximum suppression with deletions of *MNL1*, *CNE1*, and *HAC1* (0.72-fold, 0.71-fold, and 0.70-fold vs. parental control, respectively; Figure 5C,D).

### 2.5. Overexpression of ER Components Enhanced PR953 Secretion More Compared to PR961

**Figure 6:**
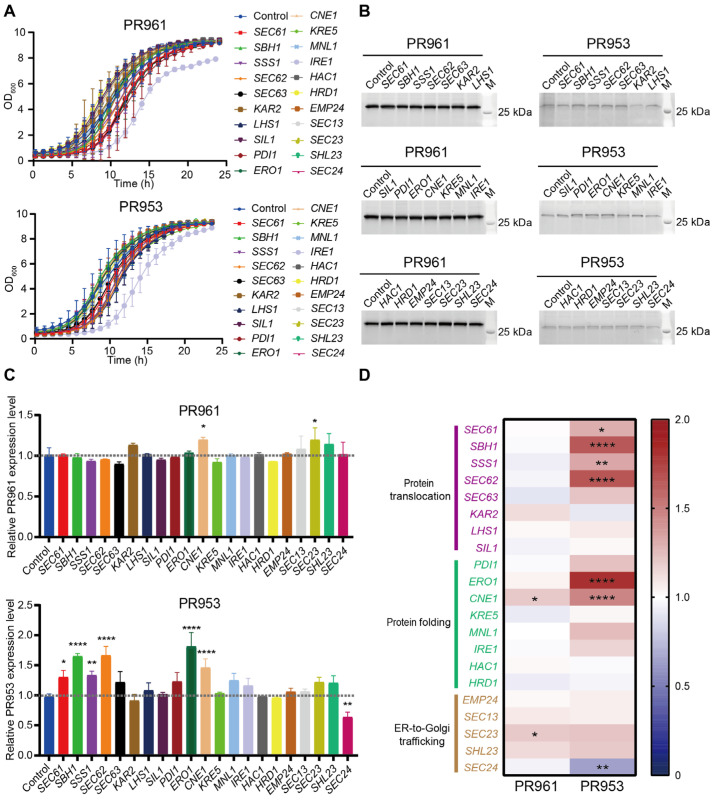
Effects on PR961 and PR953 secretion by overexpressing ER-related genes. (**A**) Growth curves of twenty-one distinct single-gene overexpression strains of PR961 and PR953 incubated 24 h in MGY medium. (**B**) SDS-PAGE analysis of PR961 and PR953 secretion levels. The gels were stained using the SYPRO dye method. (**C**) Quantification of PR961 and PR953 secretion using the ImageJ 1.53a software. Significant differences were determined from three independent experiments. (**D**) Heat map of the fold changes in PR961 and PR953 secretion relative to their respective controls. Statistical significance levels are denoted as follows: *p* < 0.05 (*), *p* < 0.01 (**), and *p* < 0.0001 (****).

We further assessed the influence of ER functional modules on the secretion of the two scFvs by overexpressing twenty-one genes, as illustrated above (Figure 4, Table S2). The growth analysis revealed that most of the overexpressed genes slightly perturbed the growth of *P. pastoris* cells without affecting biomass after 24 h of culture (Figure 6A and Figure S5B). However, the overexpression of *IRE1*, a gene related to the protein translocation module, led to a significant delay in cell growth by over 1.7-fold and a decrease in final biomass for both strains. In contrast, the deletion of *IRE1* had no effect on cell growth (Figure 5A).

Increased PR961 production was only observed with *CNE1* and *SEC23* overexpression (Figure 6B,C and Figure S6). *CNE1* is involved in the regulation of protein folding [38,39], while *SEC23* plays a role in ER-to-Golgi trafficking processes [40], highlighting the significance of folding efficiency and ER-to-Golgi transport in enhancing scFv expression in *P. pastoris*. Notably, the overexpression of six out of twenty-one genes (*SEC61*, *SBH1*, *SSS1*, *SEC62*, *ERO1*, and *CNE1*) led to significantly increased PR953 production (Figure 6C,D). These six genes are associated with two ER functional modules: *SEC61*, *SBH1*, *SSS1*, and *SEC62* are linked to translocation, and *ERO1* and *CNE1* are involved in protein folding processes. These results indicate that the translocation of the nascent PR953 peptide was impeded, leading to its relatively low secretion compared to PR961. Furthermore, the overexpression of the *SIL1*, *HAC1*, and *HRD1* genes had no effect on the secretion of both scFvs (Figure 6D), although their deletion reduced scFv production (Figure 5D).

## 3. Discussion

Our study on protein engineering in *P. pastoris* reveals a major challenge in biotechnology: sequence–structure homology is insufficient to reliably predict the efficacy of various engineering strategies on improving secretion efficiency. By testing the gene dosage, cassette architecture, and secretory pathway rewiring with two anti-SARS-CoV-2 scFv variants, we observed notable context-dependent results (Table 1), even though prior work has indicated the general efficacy of these strategies [30,31,32].

Among the three strategies, the design of the V_H_-linker-V_L_ conformation exhibited the most significant and universal optimization effect. It increased the secretion levels of PR961 and PR953 by 11.18-fold and 5.09-fold, respectively, surpassing other strategies. Our results are consistent with the previous studies [41,42]. The V_H_-linker-V_L_ conformation may improve secretion efficiency by optimizing mRNA secondary structures or facilitating the co-translational translocation of the signal peptide [43,44]. Moreover, this conformation closely follows the natural antibody folding pathway, thereby potentially reducing the risk of V_L_ domain misfolding during translation [45]. Overall, these findings propose a straightforward strategy for scFv production in *P. pastoris*.

X-ray crystal evidence demonstrates that the two scFvs, PR961 and PR953, exhibit a highly similar structure (RMSD = 0.47 Å) and share a 63% sequence homology. Notably, after 24 h of induction, both mRNA levels and the total intracellular expression of scFvs were comparable between the PR961 and PR953 strains (V_H_-linker-V_L_, two copies), with insoluble fractions constituting less than 30% of the total intracellular scFv pool in both strains (Figure S3). In contrast, the extracellular secretion levels differed by more than 12-fold between PR961 and PR953 (12.35:1 ratio; Figure 1F). These findings suggest that the marked difference in extracellular yields between the two strains primarily stems from variations in secretory efficiency rather than transcriptional or translational constraints. We hypothesized that the disparity in their secretion levels could be elucidated by investigating key components involved in protein secretory pathways [26,46]. By systematically examining twenty-one identified crucial genes with loss and overexpression, this study revealed some insights into factors influencing the secretion of the two scFvs in *P. pastoris*. Firstly, *CNE1* is the sole positive regulator for both PR961 and PR953 secretion. *CNE1* shares a 24% sequence identity with the mammalian chaperone calnexin [38], known for aiding protein folding by interacting with glycan chains [39]. Both PR961 and PR953 contained potential O-glycosylation sites. Secondly, genes involved in ER translocation play a crucial role in protein expression [47]. The seven translocation-related genes investigated in this study are essential for *P. pastoris* cell viability, and the overexpression of four of these genes significantly enhanced PR953 secretion. Interestingly, the overexpression of these genes did not affect PR961 secretion, suggesting that the secretion level of PR961 may have already reached the maximum secretion capacity of *P. pastoris*. Finally, the most notable enhancement in PR953 secretion, by 1.85-fold, was observed with the overexpression of *ERO1*. *ERO1* is associated with thiol oxidase activity, which is essential for disulfide bond formation and critical for antibody stability [36,48].

Gene dosage optimization in this study led to moderate increases in scFv production (PR961: 1.25-fold; PR953: 2.37-fold; Figure 2B,C), which were lower than the 21-fold enhancements reported for other heterologous proteins in *P. pastoris* [49]. We determined protein-specific optimal copy numbers for peak secretion levels (four copies for PR961 vs. six copies for PR953), beyond which no additional productivity improvements were observed (Figure 2). Our findings support previous studies showing that expression in *P. pastoris* is dependent on the copy number, with levels exceeding three copies failing to enhance transglutaminase secretion [50]. These results collectively indicate that the impact of gene copy number optimization on heterologous protein secretion is constrained by protein-specific thresholds, highlighting limitations in the *P. pastoris* secretion system [51]. These limitations may be attributed to ribosome flux constraints [52], where high-copy constructs lead to mRNA accumulation that overwhelms cellular translation machinery, triggering ER stress responses and inhibiting secretory efficiency.

While this study evaluated three engineering strategies for scFv production in *P. pastoris*, several limitations should be acknowledged. Firstly, the focus on two structurally homologous scFvs may limit the generalizability of these findings to other protein classes such as multi-domain enzymes or membrane proteins. Secondly, although CRISPR editing targeted twenty-one secretory pathway genes, potential interactions between modules were not explored. For example, it is unknown whether *SEC23* overexpression synergizes with *CNE1* to enhance PR953 secretion. Thirdly, beyond structural homology, the contribution of the biophysical characteristics of proteins (such as isoelectric point or surface hydrophilicity) to the secretion of heterologous proteins has not been fully explored, and further investigation is needed. Finally, in addition to genetic engineering strategies, culture conditions (including pH, dissolved oxygen, carbon/nitrogen ratios, etc.) are well-established factors influencing protein production in *P. pastoris* [53,54], but their effects were not assessed in this study and deserve further investigation.

Nevertheless, despite these limitations, our work provides a foundation for optimizing protein-specific processes. Future studies could combine multi-omics data (such as ribo-seq and ER stress sensors) with AI-driven models to understand structure–expression relationships. Furthermore, conducting the high-throughput screening of combinatorial engineering strategies may reveal synergistic effects that single-factor analyses cannot detect.

## 4. Materials and Methods

### 4.1. Strains, Plasmids, Reagents, and Media

The parental strain used in this study was *P. pastoris* GS115 (preserved in the laboratory). *E*. *coli* TOP10 (Tiangen, Beijing, China) was employed for DNA manipulation, gene cloning, and sequencing. A detailed list of all strains utilized in this study is provided in Supplementary Table S3. Plasmids pMEX9K, pTEF-PARS1, and pGAP, stored in the laboratory, were used as transformation vectors. The pMEX9K vector used in this study was constructed based on the commercial vector pPIC9K (Invitrogen, Carlsbad, CA, USA). To eliminate the *Xho* I restriction site within the kanamycin (Kana) resistance gene of pPIC9K, a synonymous mutation (CTCGAG → CTAGAG) was introduced by site-directed mutagenesis using the primer pair listed in Table S4. All restriction enzymes were purchased from New England Biolabs (Ipswich, MA, USA). Phanta Max SuperFidelity DNA polymerase, Rapid Taq DNA polymerase, and the homologous recombination kit were obtained from Vazyme Biotech (Nanjing, China). KOD DNA Polymerase was purchased from TOYOBO Biotech (Shanghai, China). Unless otherwise stated, all media components and reagents were purchased from Thermo Fisher Scientific (Waltham, MA, USA).

*E*. *coli* was grown in LB medium (0.5% yeast extract, 1% peptone, and 1% sodium chloride) at 37 °C with agitation at a speed of 250 rpm for 14 h. *P. pastoris* was grown at 30 °C in YPD medium (1% yeast extract, 2% peptone, and 2% D-glucose) on a shaker (220 rpm) for 24 h. Positive transformants were selected on YPDS (Z^+^) plates (1% yeast extract, 2% peptone, 2% dextrose, 1 mol/L sorbitol, 2% agar, and 0.1 mg/mL Zeocin) and MD plates (2% D-glucose, 1.34% YNB, and 2% agar).

### 4.2. Construction and Screening of Recombinant P. pastoris Strains

#### 4.2.1. Construction and Screening of Strains with Optimized Gene Dosage

The amino acid sequences of PR961 and PR953 were obtained from the RCSB PDB database (https://www.rcsb.org/sequence/7DET, accessed on 15 May 2023 and https://www.rcsb.org/sequence/7DEU, accessed on 15 May 2023). The complete amino acid sequence of two scFvs were back-translated to the DNA sequence, and the resulting sequences were codon optimized according to *P. pastoris* codon usage (Table S5). To facilitate the cloning procedures, sequences with *Xho* I, *Eco*R I, *Sac* I, and *Sal* I restriction sites were excluded. The final gene fragments were synthesized commercially (GENEWIZ, Beijing, China). The *PR961* and *PR953* gene fragments were amplified using specific primers. Each fragment was individually ligated into the *Xho* I-*Eco*R I sites of pMEX9K, resulting in the pMEX9K-PR961 and pMEX9K-PR953 plasmids. These recombinant plasmids were linearized with *Sac* I and subsequently integrated into the *AOX1* locus of the *P. pastoris* GS115 genome using homologous recombination and a modified electroporation transformation approach [55]. Positive clones with varying copy numbers were screened on YPD agar plates with different G418 concentrations (0.5 mg/mL, 1 mg/mL, and 2 mg/mL). The successful construction of the GS115-PR961 and GS115-PR953 strains was confirmed by PCR and DNA sequencing. All primers used in this study are listed in Supplementary Table S4.

#### 4.2.2. Construction and Screening of Strains with Different V_H_-V_L_ Orientations

The V_H_ and V_L_ fragments of scFvs were arranged on opposite ends of a (Gly 4 Ser) 3 linker in both orientations (V_H_-linker-V_L_ and V_L_-linker-V_H_). These fragments were inserted into the pMEX9K plasmid at *Xho* I-*Eco*R I sites, yielding the recombinant plasmids pMEX9K-V_H_-linker-V_L_ and pMEX9K-V_L_-linker-V_H_, respectively. Following plasmid linearization at *Sac* I, the recombinant plasmids were integrated into the *AOX1* locus of the GS115 genome by electro-transformation. Positive transformants were screened on MD plates and validated by sequencing, resulting in the establishment of recombinant strains denoted as GS115-V_H_-linker-V_L_ and GS115-V_L_-linker-V_H_.

#### 4.2.3. Construction and Screening of Gene Knockout Strains

To generate gene deletion mutants, CRISPR-Cas9 tools were employed for genetic editing. The gRNA targeting sequences are listed in Table S6. Following the amplification of the target gRNA sequence fragments using specific primers, these fragments were integrated into the pTEF vector via homologous recombination to produce the pTEF-gRNA plasmids. The pTEF-gRNA plasmid mixture, targeting the same gene, was transferred by electroporation (2000 V, 5 ms) into GS115-Cas9-PR961 or GS115-Cas9-PR953, resulting in positive transformants on YPDS (Z^+^) plates. The successful construction of the knockout strains was verified via PCR amplification and Sanger sequencing, yielding the recombinant strains GS115-PR961-gRNA and GS115-PR953-gRNA. Zeocin-resistant clones with no detectable editing events at the target locus, as confirmed by sequencing, were classified, indicating that the corresponding gRNA-targeted gene is a lethal gene for *P. pastoris*.

#### 4.2.4. Construction and Screening of Gene Over-Expression Strains

To create strains overexpressing ER secretory pathway-related genes, gene fragments were amplified from GS115 genomic DNA using specific primers. These fragments were then cloned into the pGAP vector using *Spe* I and *Xho* I sites, resulting in 21 recombinant plasmids. After linearization with *Sal* I, each plasmid was integrated into the *HIS4* locus of strains GS115-PR961 and GS115-PR953 via homologous recombination. Positive transformants were identified on MD plates, and sequencing verified the generation of recombinant GS115-PR961-gene and GS115-PR953-gene strains.

### 4.3. Evaluation of PR961 and PR953 Gene Copy Numbers by Digital PCR

The copy numbers of *PR961* and *PR953* genes were determined by droplet-digital PCR (ddPCR). The strains were cultured in YPD at 30 °C for 24 h, followed by centrifugation at 4000 rpm for 5 min to collect yeast cells for genomic DNA extraction. For quantification of the *PR961* and *PR953* genes, primers were designed to target the two scFv gene sequences and the housekeeping gene actin sequence on the genome, respectively (Table S4). The ddPCR analysis was conducted using a microfluidic sample preparation chip and a microdroplet detection chip (TARGETING ONE, Beijing, China). DNA samples were partitioned into approximately 20,000 microdroplets and subjected to PCR amplification. The PCR protocol for the microdroplet samples was as follows: an initial denaturation at 95 °C for 10 min, followed by 39 cycles of 30 s at 95 °C and 1 min at 60 °C. The fraction of positive droplets was then fitted to a Poisson distribution to determine the absolute copy number per microliter.

### 4.4. Inducible Expression and Growth Curves of Recombinant P. pastoris Strains

For protein induction, recombinant strains were inoculated into 48-well deep-well plates containing 1 mL of MGY medium (1% yeast extract, 2% peptone, 1.34% YNB, 1 mol/L potassium phosphate and 1% glycerol). Cells were cultivated for 24 h at 30 °C and with shaking at 800 rpm in a MicroScreen high-throughput microbial growth analysis system (Jieling, China). Then, cell pellets were harvested by centrifugation (2000× *g* for 5 min), resuspended in 1 mL of MMY medium (1% yeast extract, 2% peptone, 1.34% YNB and 1 mol/L potassium phosphate and 0.5% methanol), and incubated at 28 °C with shaking at 800 rpm for 24 h.

Meanwhile, cell density was monitored online every hour by measuring the optical density at 600 nm (OD_600_) using the system described above during cultivation in MGY medium. Growth curves were plotted using three replicates for each strain.

### 4.5. Quantify of Secreted Proteins with SYPRO Ruby Staining

After 24 h of induction, the cultures were centrifuged at 4000 rpm for 5 min to collect the supernatant containing secreted proteins, which were analyzed by SDS-PAGE. The OD_600_ of the yeast cultures was quantified using a UV spectrophotometer (Unico, Shanghai, China) to ensure sample uniformity. Following electrophoresis, the SDS-PAGE gels were fixed twice in a solution of 50% methanol and 10% acetic acid for 30 min each, followed by three 10 min washes in ultrapure water. For visualization, gels were stained overnight with SYPRO Ruby (Invitrogen, Eugene, OR, USA) for total protein detection. Following staining, gels were washed in buffers containing 10% methanol and 7% acetic acid for 30 min, followed by two 5 min washes in ultrapure water. Visualization was performed under excitation at 450 nm and emission at 620 nm. Finally, the intensity of the protein bands, represented as gray values, was analyzed using ImageJ 1.53a software.

### 4.6. Sequence and Structure Analysis

Amino acid sequence alignment was performed using Clustal Omega (https://www.ebi.ac.uk/Tools/msa/clustalo/, accessed on 25 July 2023) and Espript 3.0 (http://espript.ibcp.fr/ESPript/ESPript/, accessed on 25 July 2023). Potential glycosylation sites were identified using the DTU Health Tech tool (https://services.healthtech.dtu.dk/, accessed on 20 March 2024). The crystal structures of PR961 (PDB ID: 7DET) and PR953 (PDB ID: 7DEU) were obtained from the RCSB PDB database (https://www.rcsb.org/, accessed on 15 May 2023).

### 4.7. Statistical Analysis

All data from three independent experiments were analyzed using GraphPad Prism software version 8 (Graph Pad Software Inc., San Diego, CA, USA), and are presented as the mean ± standard deviation (SD). A student’s *t*-test was used to compare basal secretion levels between PR961 and PR953 parental control strains. A one-way analysis of variance (ANOVA) was applied to assess the variations in secretion levels across groups of strains subjected to identical engineering strategies. A *p* value of less than 0.05 was considered significant.

## Figures and Tables

**Table 1:** The significant fold change in PR961 and PR953 secretion under different engineering strategies in *P. pastoris*.

Engineering Strategy				Fold Change Engineered vs. Parental	
				PR961	PR953
gene dosage optimization	2			-	-
	4			1.25 * ± 0.14	-
	5			ns	2.18 * ± 0.25
	6			-	2.37 * ± 0.06
	7			-	2.30 * ± 0.10
cassette architecture engineering	V_L_-linker-V_H_			-	-
	V_H_-linker-V_L_			11.18 * ± 0.37	5.09 * ± 0.43
ER secretory pathway reprogramming	Protein translocation	*SEC61*	+	ns	1.30 * ± 0.11
		*SBH1*	+	ns	1.65 * ± 0.05
		*SSS1*	+	ns	1.33 * ± 0.07
		*SEC62*	+	ns	1.66 * ± 0.15
		*SIL1*	-	0.83 * ± 0.03	0.70 * ± 0.05
	Protein folding	*ERO1*	+	ns	1.81 * ± 0.23
		*CNE1*	+	1.20 * ± 0.03	1.46 * ± 0.15
			-	0.71 * ± 0.01	0.48 * ± 0.03
		*KRE5*	-	ns	0.60 * ± 0.04
		*MNL1*	-	0.72 * ± 0.01	ns
		*IRE1*	-	0.76 * ± 0.02	ns
		*HAC1*	-	0.70 * ± 0.03	0.75 * ± 0.05
		*HRD1*	-	0.91 * ± 0.01	0.52 * ± 0.01
	ER-to-Golgi trafficking	*SEC23*	+	1.20 * ± 0.15	ns
		*SEC24*	+	ns	0.64 * ± 0.08

Data are represented as mean fold change (relative to respective parental control group) ±SEM (n = 3 biological replicates/group). Statistical significance is denoted as *p* ≤ 0.05 (*), *p* > 0.05 (ns). +: Overexpression of genes. -: Knockout of genes.

## Data Availability

The raw data supporting the conclusions of this article will be made available by the authors, without undue reservation.

## Supplementary Materials

The following supporting information can be downloaded at: https://www.mdpi.com/article/10.3390/ijms26104922/s1. Reference [56] is cited in the supplementary materials.

## Abbreviations

The following abbreviations are used in this manuscript:

ER	Endoplasmic reticulum
scFv	Single-chain variable fragment
CRISPR	Clustered regularly interspaced short palindromic repeats
Pdi	Protein disulfide isomerase
UPR	Unfolded protein reaction
SDS-PAGE	Sodium dodecyl sulfate-polyacrylamide gel electrophoresis
V_L_	Variable light chain
V_H_	Variable heavy chain
Cas9	CRISPR-associated protein 9
ERAD	Endoplasmic reticulum-associated degradation
ddPCR	Droplet-digital PCR

## References

[1] Radziwon K., Weeks A.M. (2021). Protein engineering for selective proteomics. Curr. Opin. Chem. Biol..

[2] Lakhawat S.S., Chandel S., Jaswal S.K., Sharma P.K. (2023). Protein Engineering, a Robust Tool to Engineer Novel Functions in Protein. Protein Pept. Lett..

[3] Meinen B.A., Bahl C.D. (2021). Breakthroughs in computational design methods open up new frontiers for de novo protein engineering. Protein Eng. Des. Sel..

[4] Linsky T.W., Noble K., Tobin A.R., Crow R., Carter L., Urbauer J.L., Baker D., Strauch E.-M. (2022). Sampling of structure and sequence space of small protein folds. Nat. Commun..

[5] Orsi E., Schada von Borzyskowski L., Noack S., Nikel P.I., Lindner S.N. (2024). Automated in vivo enzyme engineering accelerates biocatalyst optimization. Nat. Commun..

[6] Nammi B., Jayasinghe-Arachchige V.M., Madugula S.S., Artiles M., Radler C.N., Pham T., Liu J., Wang S. (2025). CasGen: A Regularized Generative Model for CRISPR Cas Protein Design with Classification and Margin-Based Optimization. bioRxiv.

[7] Moraes L.M.P.d., Marques H.F., Reis V.C.B., Coelho C.M., Leitão M.d.C., Galdino A.S., Porto de Souza T.P., Piva L.C., Perez A.L.A., Trichez D. (2024). Applications of the Methylotrophic Yeast *Komagataella phaffii* in the Context of Modern Biotechnology. J. Fungi.

[8] Karbalaei M., Rezaee S.A., Farsiani H. (2020). Pichia pastoris: A highly successful expression system for optimal synthesis of heterologous proteins. J. Cell. Physiol..

[9] Barone G.D., Emmerstorfer-Augustin A., Biundo A., Pisano I., Coccetti P., Mapelli V., Camattari A. (2023). Industrial Production of Proteins with *Pichia pastoris*—*Komagataella phaffii*. Biomolecules.

[10] Wang P., Huang L., Jiang H., Tian J., Chu X., Wu N. (2014). Improving the secretion of a methyl parathion hydrolase in *Pichia pastoris* by modifying its N-terminal sequence. PLoS ONE.

[11] Zhao F., Yu C.-h., Liu Y. (2017). Codon usage regulates protein structure and function by affecting translation elongation speed in Drosophila cells. Nucleic Acids Res..

[12] Sun Z., Guerriero C.J., Brodsky J.L. (2021). Substrate ubiquitination retains misfolded membrane proteins in the endoplasmic reticulum for degradation. Cell Rep..

[13] Delic M., Valli M., Graf A.B., Pfeffer M., Mattanovich D., Gasser B. (2013). The secretory pathway: Exploring yeast diversity. FEMS Microbiol. Rev..

[14] Werten M.W., Eggink G., Stuart M.A.C., de Wolf F.A. (2019). Production of protein-based polymers in *Pichia pastoris*. Biotechnol. Adv..

[15] Suarez S., Martiny A., Asempa T. (2021). Gene amplification uncovers large previously unrecognized cryptic antibiotic resistance potential in *E. coli*, Microbiol. Spectrum.

[16] Zheng X., Zhang Y., Liu X., Li C., Lin Y., Liang S. (2020). High-level expression and biochemical properties of a thermo-alkaline pectate lyase from *Bacillus* sp. RN1 in *Pichia pastoris* with potential in ramie degumming. Front. Bioeng. Biotechnol..

[17] Wang X., Goldstein D.B. (2020). Enhancer domains predict gene pathogenicity and inform gene discovery in complex disease. Am. J. Hum. Genet..

[18] Bakoulis S., Krautz R., Alcaraz N., Salvatore M., Andersson R. (2022). Endogenous retroviruses co-opted as divergently transcribed regulatory elements shape the regulatory landscape of embryonic stem cells. Nucleic Acids Res..

[19] Ben Azoun S., Belhaj A.E., Göngrich R., Gasser B., Kallel H. (2016). Molecular optimization of rabies virus glycoprotein expression in *Pichia pastoris*. Microb. Biotechnol..

[20] Datta S. (2021). Optimizing Granulocyte Colony-Stimulating Factor Transcript for Enhanced Expression in *Escherichia coli*. Front. Bioeng. Biotechnol..

[21] Gidalevitz T., Stevens F., Argon Y. (2013). Orchestration of secretory protein folding by ER chaperones. Biochim. Biophys. Acta (BBA) Mol. Cell Res..

[22] Du H., Zheng C., Aslam M., Xie X., Wang W., Yang Y., Liu X. (2021). Endoplasmic reticulum-mediated protein quality control and endoplasmic reticulum-associated degradation pathway explain the reduction of N-glycoprotein level under the lead stress. Front. Plant Sci..

[23] Strasser R. (2018). Protein quality control in the endoplasmic reticulum of plants. Annu. Rev. Plant Biol..

[24] Hou J., Tyo K.E.J., Liu Z., Petranovic D., Nielsen J. (2012). Metabolic engineering of recombinant protein secretion by *Saccharomyces cerevisiae*. FEMS Yeast Res..

[25] Raschmanová H., Weninger A., Knejzlík Z., Melzoch K., Kovar K. (2021). Engineering of the unfolded protein response pathway in Pichia pastoris: Enhancing production of secreted recombinant proteins. Appl. Microbiol. Biotechnol..

[26] Zhang Y.-S., Gong J.-S., Jiang J.-Y., Xu Z.-H., Shi J.-S. (2024). Engineering protein translocation and unfolded protein response enhanced human PH-20 secretion in *Pichia pastoris*. Appl. Microbiol. Biotechnol..

[27] Zahrl R.J., Prielhofer R., Ata Ö., Baumann K., Mattanovich D., Gasser B. (2022). Pushing and pulling proteins into the yeast secretory pathway enhances recombinant protein secretion. Metab. Eng..

[28] Fu D., Zhang G., Wang Y., Zhang Z., Hu H., Shen S., Wu J., Li B., Li X., Fang Y. (2021). Structural basis for SARS-CoV-2 neutralizing antibodies with novel binding epitopes. PLoS Biol..

[29] Karlsson J., Larsson E. (2016). FocalScan: Scanning for altered genes in cancer based on coordinated DNA and RNA change. Nucleic Acids Res..

[30] Tang X., Kadara H., Behrens C., Liu D.D., Xiao Y., Rice D., Gazdar A.F., Fujimoto J., Moran C., Varella-Garcia M. (2011). Abnormalities of the TITF-1 lineage-specific oncogene in NSCLC: Implications in lung cancer pathogenesis and prognosis. Clin. Cancer Res..

[31] Bezrookove V., De Semir D., Nosrati M., Tong S., Wu C., Thummala S., Dar A.A., Leong S.P., Cleaver J.E., Sagebiel R.W. (2014). Prognostic impact of PHIP copy number in melanoma: Linkage to ulceration. J. Investig. Dermatol..

[32] Satheeshkumar P.K. (2020). Expression of single chain variable fragment (scFv) molecules in plants: A comprehensive update. Mol. Biotechnol..

[33] Liu C., Gong J.-S., Su C., Li H., Li H., Rao Z.-M., Xu Z.-H., Shi J.-S. (2022). Pathway engineering facilitates efficient protein expression in *Pichia pastoris*. Appl. Microbiol. Biotechnol..

[34] Santiago-Tirado F.H., Hurtaux T., Geddes-McAlister J., Nguyen D., Helms V., Doering T.L., Römisch K. (2023). The ER protein translocation channel subunit Sbh1 controls virulence of *Cryptococcus neoformans*. mBio.

[35] Zimmermann J.S., Linxweiler J., Radosa J.C., Linxweiler M., Zimmermann R. (2022). The endoplasmic reticulum membrane protein Sec62 as potential therapeutic target in SEC62 overexpressing tumors. Front. Physiol..

[36] Frand A.R., Kaiser C.A. (1998). The ERO1 gene of yeast is required for oxidation of protein dithiols in the endoplasmic reticulum. Mol. Cell.

[37] Esaki M., Liu Y., Glick B.S. (2006). The budding yeast Pichia pastoris has a novel Sec23p homolog. FEBS Lett..

[38] Parlati F., Dominguez M., Bergeron J.J.M., Thomas D.Y. (1995). *Saccharomyces cerevisiae* CNE1 Encodes an Endoplasmic Reticulum (ER) Membrane Protein with Sequence Similarity to Calnexin and Calreticulin and Functions as a Constituent of the ER Quality Control Apparatus. J. Biol. Chem..

[39] Kozlov G., Gehring K. (2020). Calnexin cycle–structural features of the ER chaperone system. FEBS J..

[40] Jing J., Wang B., Liu P. (2019). The functional role of SEC23 in vesicle transportation, autophagy and cancer. Int. J. Biol. Sci..

[41] Bujotzek A., Dunbar J., Lipsmeier F., Schäfer W., Antes I., Deane C.M., Georges G. (2015). Prediction of VH–VL domain orientation for antibody variable domain modeling. Proteins Struct. Funct. Bioinform..

[42] Liu A., Ye Y., Chen W., Wang X., Chen F. (2015). Expression of VH-linker-VL orientation-dependent single-chain Fv antibody fragment derived from hybridoma 2E6 against aflatoxin B1 in *Escherichia coli*. J. Ind. Microbiol. Biotechnol..

[43] Kozak M. (1980). Influence of mRNA secondary structure on binding and migration of 40S ribosomal subunits. Cell.

[44] Kim Y., Lee G., Jeon E., Sohn E.j., Lee Y., Kang H., Lee D.w., Kim D.H., Hwang I. (2013). The immediate upstream region of the 5′-UTR from the AUG start codon has a pronounced effect on the translational efficiency in *Arabidopsis thaliana*. Nucleic Acids Res..

[45] Simpson E.R., Herold E.M., Buchner J. (2009). The folding pathway of the antibody VL domain. J. Mol. Biol..

[46] Besada-Lombana P.B., Da Silva N.A. (2019). Engineering the early secretory pathway for increased protein secretion in *Saccharomyces cerevisiae*. Metab. Eng..

[47] Ellgaard L., McCaul N., Chatsisvili A., Braakman I. (2016). Co-and post-translational protein folding in the ER. Traffic.

[48] Sevier C.S., Kaiser C.A. (2008). Ero1 and redox homeostasis in the endoplasmic reticulum. Biochim. Biophys. Acta (BBA) Mol. Cell Res..

[49] Chen X., Deng J., Zheng X., Zhang J., Zhou Z., Wei H., Zhan C.-G., Zheng F. (2019). Development of a long-acting Fc-fused cocaine hydrolase with improved yield of protein expression. Chem. Biol. Interact..

[50] Song X., Shao C., Guo Y., Wang Y., Cai J. (2019). Improved the expression level of active transglutaminase by directional increasing copy of mtg gene in *Pichia pastoris*. BMC Biotechnol..

[51] Xie S., Sun S., Lin F., Li M., Pu Y., Cheng Y., Xu B., Liu Z., da Costa Sousa L., Dale B.E. (2019). Mechanism-Guided Design of Highly Efficient Protein Secretion and Lipid Conversion for Biomanufacturing and Biorefining. Adv. Sci..

[52] Proshkin S., Rahmouni A.R., Mironov A., Nudler E. (2010). Cooperation between translating ribosomes and RNA polymerase in transcription elongation. Science.

[53] Joseph J.A., Akkermans S., Van Impe J.F. (2022). Effects of temperature and pH on recombinant Thaumatin II production by *Pichia pastoris*. Foods.

[54] Li Z., Xiong F., Lin Q., d’Anjou M., Daugulis A.J., Yang D.S., Hew C.L. (2001). Low-temperature increases the yield of biologically active herring antifreeze protein in *Pichia pastoris*. Protein Expr. Purif..

[55] Faber K.N., Haima P., Harder W., Veenhuis M., AB G. (1994). Highly-efficient electrotransformation of the yeast *Hansenula polymorpha*. Curr. Genet..

[56] Besleaga M., Vignolle G.A., Kopp J., Spadiut O., Mach R.L., Mach-Aigner A.R., Zimmermann C. (2023). Evaluation of reference genes for transcript analyses in *Komagataella phaffii* (*Pichia pastoris*). Fungal Biol. Biotechnol..

