# Supplementary material for: Structural Homology Fails to Predict Secretion Efficiency in Pichia pastoris: Divergent Responses of Architecturally Similar scFvs to Multi-Parametric Genetic Engineering
Source: Int J Mol Sci. 2025 May 21;26(10):4922. doi: 10.3390/ijms26104922 (PMC12120847; doi:10.3390/ijms26104922)
Supplement: Supplementary file 1 [file ijms-26-04922-s001.zip › ijms-3574789-supplementary.pdf]

SUPPLEMENTARY METHODS

RNA isolation and quantitative real-time PCR

Cell pellets were harvested by centrifugation (2000 g, 5 minutes) 0 and 24 hours after induction. After lysing yeast cells with lysis buffer (20 U lyticase (Tiangen), 1 M sorbitol, 0.1 M EDTA, 0.2% 2 M DTT, and 0.1% β-mercaptoethanol), total RNAs of samples were isolated using the RNA extraction kit (Tiangen Biotech, Beijing, China). Then, 800 ng of RNA per sample was reverse transcribed to cDNA using the PrimeScript™ RT reagent kit (Takara, Japan) according to the manufacturer's instructions. Quantitative real-time PCR (qPCR) was performed using the TB Green® Premix Ex Taq™ II (Tli RNase H Plus) (TaKaRa, Japan) on the CFX Opus 96 Real-Time PCR System (BIO-RAD, USA). The qRT-PCR reaction program was performed under the following conditions: 95 °C for 30 s followed by 40 cycles of 95 °C for 5 s and 60 °C for 30 s. The experiments were performed in triplicate, and the values were normalized to that of *RSC* [1]. The primer sequences are listed in Table S4.

Quantify of intracellular proteins with Western blot

Cell pellets were harvested by centrifugation (2000 g, 5 minutes) 24 hours after induction. After being washed three times with PBS, 2×10^8 *P. pastoris* cells were lysed in 100 μL lysis buffer and incubated at 30℃ and 220 rpm for 60 minutes. The lysis buffer consisted of 20 U lyticase (Tiangen, Beijing, China) and a mixed buffer. The mixed buffer contained 1×PBS, 1×cocktail (Thermo Scientific, USA), 1 mM PMSF (Sigma-Aldrich, DEU), 5 mM EDTA, and 20 mM DTT. After centrifugation (13,000 rpm, 4°C, 10 minutes), the supernatant and the pellet were separated. The supernatant (soluble fraction) was collected and mixed with 25 μL of 5×loading buffer, and the pellet (insoluble fraction) was resuspended in 100 μL of mixed buffer mixed with 25 μL of 5×loading buffer. The mixtures were then boiled at 100°C for 10 minutes. Samples (30 μL) were separated by sodium dodecyl sulfate-polyacrylamide gel electrophoresis (SDS-PAGE) and transferred to polyvinylidene fluoride (PVDF) membranes. The membrane was blocked with 5% skimmed milk in 1×TBST for 2 hours at room temperature and then incubated with an anti-(G4S)n (B02H1)mAb(HRP) antibody (1:1000 dilution; Hycells, Wuhan, China) (molecular weight: 26.4 kDa) overnight at 4°C. We used β-tubulin (T1100HRP, LABLEAD) as a housekeeping protein. The PVDF membranes were washed three times with TBST (10 minutes each time). After washing, the immunoreactive bands on the membrane were visualized using the enhanced chemiluminescence (ECL) kit (Beyotime, Shanghai, China). Quantification of protein bands was performed using the ImageJ software and values were normalized to tubulin for each sample.

BLI binding affinity measurement

Binding kinetics and affinity of scFvs to the SARS-CoV-2 RBD were measured by biolayer interferometry (BLI) using Octet R8. After 24 h of induction, the supernatant was collected by centrifugation (2000 g, 5 minutes) and the protein concentration was determined via BCA Protein Assay Kit (Thermo Fisher, USA). The protein A biosensor (Sartorius) was pre-balanced in 200 μL kinetics buffer (1×PBS with 0.02% Tween-20) for 30 minutes, and then transferred to 200 μL RBD protein solution at a concentration of 2 μg/mL for 2 minutes. The biosensor was then washed in the kinetics buffer to remove unbound RBD. After that, the biosensor was placed in the wells of a 96-well plate with 200 μL of scFv samples for 2 minutes. To remove nonspecific binding, the biosensor was washed in kinetics buffer for another minute. Data analysis was conducted using Octet Analysis software version 12.2 (Sartorius). Background binding was measured in wells without proteins and subtracted from the data. Sensorgrams were fitted with a 1:1 binding model to calculate kinetic parameters such as the binding rate constant (Kon), dissociation rate constant (kdis), and equilibrium dissociation constant (KD).

Table S1. The binding kinetic constants and associated errors of PR961 and PR953 combined with RBDs

Recombinant strain	KD(M)	Kon(1/Ms)	Kon Error	kdis (1/s)	kdis Error
PR961-V_H-linker-V_L	5.09E-08±3.95E-08	7.08E04±1.54E04	1.30E03±6.34E01	3.20E-03±1.69E-03	3.83E-05±1.57E-05
PR961-V_L-linker-V_H	1.23E-06±3.50E-07	1.66E04±2.61E03	4.40E02±9.43E01	2.04E-02±6.64E-03	2.00E-04±6.51E-05
PR953-V_H-linker-V_L	1.50E-06±8.77E-07	4.52E03±3.28E03	8.48E01±7.21E01	4.94E-03±1.80E-03	3.75E-05±2.49E-05
PR953-V_L-linker-V_H	3.42E-07±5.83E-08	1.65E04±9.71E02	1.73E02±2.72E01	5.60E-03±6.61E-04	4.18E-05±3.34E-06

Table S2. Three ER functional modules and twenty-one targeted genes in this study

ER functional modules	Gene name	Uniprot ID
Protein translocation	*SEC61**	**C4QVJ2**
	*SBH1**	**C4R2I3**
	*SSS1**	**C4QVV4**
	*SEC62**	**C4R689**
	*SEC63**	**C4R7S0**
	*KAR2**	**C4QZS3**
	*LHS1**	**C4QV45**
	SIL1	**C4QWI3**
Protein folding	*PDI1*	**C4R938**
	*ERO1**	**C4QVU1**
	CNE1	**C4R0B4**
	KRE5	**C4R603**
	MNL1	**C4R5W2**
	IRE1	**C4R6I4**
	HAC1	**C4QWY5**
	HRD1	**C4R706**
ER-to-Golgi trafficking	*EMP24*	**C4R1A2**
	*SEC13**	**P53024**
	*SEC23**	**C4R8D5**
	*SHL23**	**C4QVY3**
	*SEC24**	**C4R525**

*: lethal genes.

Table S3. Strains and plasmids used in this study

Strains and plasmids	Functions or relevant characteristics	Source
GS115	Expression host bacteria, Wild type	preserved in the laboratory
GS115/ Cas9	Express the Cas9 protein	This study
Escherichia coli Top10	Cloning host bacteria, F^- *mcr*AΔ (*mrr-hsd* RMS-*mcr* BC) φ*80lac*ZΔM15Δ*lac*X74 *rec*A1 *ara*Δ139Δ (*ara-leu*) 7697*gal*U *gal*K *rps*L (Str^r) *end*A1 *nup*G	Tiangen
pMEX9K	Cloning vector of the target gene, *ori*, PpHIS4, Amp^r, Kan^r, *AOX1* promoter, α-factor, *AOX1* terminator	This study
pMEX9K-PR961	PR961 expression vector	pMEX9K-PR961
pMEX9K-PR953	PR953 expression vector	pMEX9K-PR953
pTEF-PARS1	The gRNA cloning vector, *PARS1*, pUC *ori*, Zeo^r, TEF1 promoter, *AOX1* terminator	preserved in the laboratory
pTEF-*SIL1*-gRNA1	The gRNA1 cloning vector targeting *SIL1*	This study
pTEF-*SIL1*-gRNA2	The gRNA2 cloning vector targeting *SIL1*	This study
pTEF-*SIL1*-gRNA3	The gRNA3 cloning vector targeting *SIL1*	This study
pTEF-*PDI1*-gRNA1	The gRNA1 cloning vector targeting *PDI1*	This study
pTEF-*PDI1*-gRNA2	The gRNA2 cloning vector targeting *PDI1*	This study
pTEF-*PDI1*-gRNA3	The gRNA3 cloning vector targeting *PDI1*	This study
pTEF-*CNE1*-gRNA1	The gRNA1 cloning vector targeting *CNE1*	This study
pTEF-*CNE1*-gRNA2	The gRNA2 cloning vector targeting *CNE1*	This study

Strains and plasmids	Functions or relevant characteristics	Source
pTEF-*KRE5*-gRNA1	The gRNA1 cloning vector targeting *KRE5*	This study
pTEF-*KRE5*-gRNA2	The gRNA2 cloning vector targeting *KRE5*	This study
pTEF-*KRE5*-gRNA3	The gRNA3 cloning vector targeting *KRE5*	This study
pTEF-*MNL1*-gRNA1	The gRNA1 cloning vector targeting *MNL1*	This study
pTEF-*MNL1*-gRNA2	The gRNA2 cloning vector targeting *MNL1*	This study
pTEF-*MNL1*-gRNA3	The gRNA3 cloning vector targeting *MNL1*	This study
pTEF-*IRE1*-gRNA1	The gRNA1 cloning vector targeting *IRE1*	This study
pTEF-*IRE1*-gRNA2	The gRNA2 cloning vector targeting *IRE1*	This study
pTEF-*HAC1*-gRNA1	The gRNA1 cloning vector targeting *HAC1*	This study
pTEF-*HAC1*-gRNA2	The gRNA2 cloning vector targeting *HAC1*	This study
pTEF-*HRD1*-gRNA1	The gRNA1 cloning vector targeting *HRD1*	This study
pTEF-*HRD1*-gRNA2	The gRNA2 cloning vector targeting *HRD1*	This study
pTEF-*EMP24*-gRNA1	The gRNA1 cloning vector targeting *EMP24*	This study
pTEF-*EMP24*-gRNA2	The gRNA2 cloning vector targeting *EMP24*	This study
pTEF-*EMP24*-gRNA3	The gRNA3 cloning vector targeting *EMP24*	This study
pGAP	Cloning vector of the target gene, *ori*, PpHIS4, Amp^r, GAP1 promoter, CYC1 terminator	preserved in the laboratory
pGAP-Sec61	Sec61 protein expression vector	This study
pGAP-Sbh1	Sbh1 protein expression vector	This study
pGAP-Sss1	Sss1 protein expression vector	This study
pGAP-Sec62	Sec62 protein expression vector	This study
pGAP-Sec63	Sec63 protein expression vector	This study
pGAP-Kar2	Kar2 protein expression vector	This study
pGAP-Lhs1	Lhs1 protein expression vector	This study
pGAP-Sil1	Sil1 protein expression vector	This study
pGAP-Pdi1	Pdi1 protein expression vector	This study
pGAP-Ero1	Ero1 protein expression vector	This study
pGAP-Kre5	Kre5 protein expression vector	This study
pGAP-Mnl1	Mnl1 protein expression vector	This study
pGAP-Ire1	Ire1 protein expression vector	This study
pGAP-Hac1	Hac1 protein expression vector	This study
pGAP-Hrd1	Hrd1 protein expression vector	This study
pGAP-Emp24	Emp24 protein expression vector	This study
pGAP-Sec12	Sec12 protein expression vector	This study
pGAP-Sec23	Sec23 protein expression vector	This study
pGAP-Shl23	Shl23 protein expression vector	This study
pGAP-Sec24	Sec24 protein expression vector	This study

Table S4. Primers used in this study

Primers	Sequences (5′→3′)	Source
5306U18	TACGCGATCGCTGTTAAA	This study
6057L15	GAGGCCGTTGAGCAC	This study
Muxho5	TGCTCTAGGCCGCGATTA	This study
Muxho3	AGACGTTTCCCGTTGAAT	This study

Primers	Sequences (5′→3′)	Source
PCgR-F	AGTAAGCTCGTCTCAGATCTTAAG	This study
PCgF-F	CTAATCTAAGGGGCGATCTG	This study
PCg-R	TGGCATTCTGACATCCTCTTGAG	This study
GAP1-F	CGTCGCTGGCAATAATAGCG	This study
CYC1-R	CCTTCCTTTTCGGTTAGAGC	This study
5′AOX	GACTGGTTCCAATTGACAAGC	GENEWIZ
3′AOX	GCAAATGGCATTCTGACATCC	GENEWIZ
dPR961-F	GGTGGAGGTGGATCTGATATTG	This study
dPR961-R	ACAGATTCAGAAGCTCTACAAGTAA	This study
dPR961-P	TCCTGCTTCTTTGGCTGTTTCTCCT	This study
dPR953-F	TGGTTAAACCTGGTGCTTCT	This study
dPR953-R	CCACCGATCCATTCCAAAGA	This study
dPR953-P	TGCATTGGGTTAAACAATCTCATGGT	This study
dPR961-L-F	TGAGATCTGAAGATACTGCTGTTTA	This study
dPR961-L-R	AGAAACAGTAACCAAAGTACCTCTAC	This study
dPR961-L-P	TGTGCTAGAGCTGATGGTTATGAATGGT	This study
dPR953-L-F	GGAAATTAAAGGAGGTGGAGGA	This study
dPR953-L-R	GCACCAGGTTTAACCAATTCAG	This study
dPR953-L-P	AGGTTCTGGTGGAGGTGGATCTGA	This study
dactin-F	GTATGTGTAAGGCCGGATAC	This study
dactin-R	CTTGGTCTACCTACGACCG	This study
dactin-P	CCGGAGACGACGCCCCACAC(5′-VIC,3′-BHQ1)	This study
qPR961-F	TTCTTCTGGTGGTGGTGGTT	This study
qPR961-R	TGACCAGGAGAAACAGCCAA	This study
qPR953-F	TGGAGGTGGAGGATCTGGTG	This study
qPR953-R	ACCAAGCAACATTAGTACCAACA	This study
qRSC-F	CACGATGCCTGCTGATGTGG	This study
qRSC-R	GGGTACATACACTGGAGGACCTC	This study
SIL1-g1-F	AGGACGAAACGAGTAAGCTCGTCTCAGATCGTTAGAATCGTTTGTTCGGA	This study
SIL1-g1-R	ATTTTAACTTGCTATTTCTAGCTCTAAAACTCCGAACAAACGATTCTAAC	This study
SIL1-g2-F	AGGACGAAACGAGTAAGCTCGTCTCAGATCGTTAGCTATTGCCTCCCAAT	This study
SIL1-g2-R	ATTTTAACTTGCTATTTCTAGCTCTAAAACATTGGGAGGCAATAGCTAAC	This study
SIL1-g3-F	AGGACGAAACGAGTAAGCTCGTCTCAGATCAACAAACGATTCTAACCAAT	This study
SIL1-g3-R	ATTTTAACTTGCTATTTCTAGCTCTAAAACATTGGTTAGAATCGTTTGTT	This study
PDI1-g1-F	AGGACGAAACGAGTAAGCTCGTCTCAGATCCAACTGGAATATTAAAACTG	This study
PDI1-g1-R	ATTTTAACTTGCTATTTCTAGCTCTAAAACCAGTTTTAATATTCCAGTTG	This study
PDI1-g2-F	AGGACGAAACGAGTAAGCTCGTCTCAGATCTGCTTGTGCTAGTGTGAGAG	This study
PDI1-g2-R	ATTTTAACTTGCTATTTCTAGCTCTAAAACCTCTCACACTAGCACAAGCA	This study
PDI1-g3-F	AGGACGAAACGAGTAAGCTCGTCTCAGATCACTAGCACAAGCAAGTGATC	This study
PDI1-g3-R	ATTTTAACTTGCTATTTCTAGCTCTAAAACGATCACTTGCTTGTGCTAGT	This study
CNE1-g1-F	AGGACGAAACGAGTAAGCTCGTCTCAGATCGGGTCTGAACTCAGCGGGTT	This study
CNE1-g1-R	ATTTTAACTTGCTATTTCTAGCTCTAAAACAACCCGCTGAGTTCAGACCC	This study
CNE1-g2-F	AGGACGAAACGAGTAAGCTCGTCTCAGATCGAACTCAGCGGGTTCGGATT	This study
CNE1-g2-R	ATTTTAACTTGCTATTTCTAGCTCTAAAACAATCCGAACCCGCTGAGTTC	This study
KRE5-g1-F	AGGACGAAACGAGTAAGCTCGTCTCAGATCATATCAGTTTGAAGGCCAAT	This study
KRE5-g1-R	ATTTTAACTTGCTATTTCTAGCTCTAAAACATTGGCCTTCAAACTGATAT	This study
KRE5-g2-F	AGGACGAAACGAGTAAGCTCGTCTCAGATCGTTTGTTGATATCAGTTTGA	This study
KRE5-g2-R	ATTTTAACTTGCTATTTCTAGCTCTAAAACTCAAACTGATATCAACAAAC	This study

Primers	Sequences (5′→3′)	Source
KRE5-g3-F	AGGACGAAACGAGTAAGCTCGTCTCAGATCTCCTTTCCCACTGTTACTAC	This study
KRE5-g3-R	ATTTTAACTTGCTATTTCTAGCTCTAAAACGTAGTAACAGTGGGAAAGGA	This study
MNL1-g1-F	AGGACGAAACGAGTAAGCTCGTCTCAGATCGAGACATACCCACATCTAAT	This study
MNL1-g1-R	ATTTTAACTTGCTATTTCTAGCTCTAAAACATTAGATGTGGGTATGTCTC	This study
MNL1-g2-F	AGGACGAAACGAGTAAGCTCGTCTCAGATCTGTCTCTAGGTACTCTGATG	This study
MNL1-g2-R	ATTTTAACTTGCTATTTCTAGCTCTAAAACCATCAGAGTACCTAGAGACA	This study
MNL1-g3-F	AGGACGAAACGAGTAAGCTCGTCTCAGATCAGCTGCTCAAGAATGAGACG	This study
MNL1-g3-R	ATTTTAACTTGCTATTTCTAGCTCTAAAACCGTCTCATTCTTGAGCAGCT	This study
IRE1-g1-F	AGGACGAAACGAGTAAGCTCGTCTCAGATCATCTGCGTTTACTCATAATG	This study
IRE1-g1-R	ATTTTAACTTGCTATTTCTAGCTCTAAAACCATTATGAGTAAACGCAGAT	This study
IRE1-g2-F	AGGACGAAACGAGTAAGCTCGTCTCAGATCGCAGAGCTTCCTTGTTATAC	This study
IRE1-g2-R	ATTTTAACTTGCTATTTCTAGCTCTAAAACGTATAACAAGGAAGCTCTGC	This study
HAC1-g1-F	AGGACGAAACGAGTAAGCTCGTCTCAGATCAGCAAAGACGGAAGAAGAAA	This study
HAC1-g1-R	ATTTTAACTTGCTATTTCTAGCTCTAAAACTTTCTTCTTCCGTCTTTGCT	This study
HAC1-g2-F	AGGACGAAACGAGTAAGCTCGTCTCAGATCTATCCTACGTAATAGGAGAG	This study
HAC1-g2-R	ATTTTAACTTGCTATTTCTAGCTCTAAAACCTCTCCTATTACGTAGGATA	This study
HRD1-g1-F	AGGACGAAACGAGTAAGCTCGTCTCAGATCCAGGATCCGAAGCCAAAATA	This study
HRD1-g1-R	CAGGATCCGAAGCCAAAATAGTTTTAGAGCTAGAAATAGCAAGTTAAAAT	This study
HRD1-g2-F	AGGACGAAACGAGTAAGCTCGTCTCAGATCTGATATCCATATTTTGGCTT	This study
HRD1-g2-R	TGATATCCATATTTTGGCTTGTTTTAGAGCTAGAAATAGCAAGTTAAAAT	This study
EMP24-g1-F	AGGACGAAACGAGTAAGCTCGTCTCAGATCTTCACTATTCTGCTACTCTT	This study
EMP24-g1-R	ATTTTAACTTGCTATTTCTAGCTCTAAAACAAGAGTAGCAGAATAGTGAA	This study
EMP24-g2-F	AGGACGAAACGAGTAAGCTCGTCTCAGATCAAACATTCAATTCCATGCGG	This study
EMP24-g2-R	ATTTTAACTTGCTATTTCTAGCTCTAAAACCCGCATGGAATTGAATGTTT	This study
EMP24-g3-F	AGGACGAAACGAGTAAGCTCGTCTCAGATCAAAAAACATTCAATTCCATG	This study
EMP24-g3-R	ATTTTAACTTGCTATTTCTAGCTCTAAAACCATGGAATTGAATGTTTTTT	This study
gSIL1-F	AAGTAGCACAGACGTTCCAGAAGAG	This study
gSIL1-R	ATCATCAGCAAACGAACCGGTGGAATC	This study
gPDI1-F	TCTATTGTCTTACGCGGCTCTTTAG	This study
gPDI1-R	TAGCCTTGACATAATTCCTTCTCC	This study
gCNE1-F	TCTGTTTCATCCCCCAACTGCACTC	This study
gCNE1-R	TTGGGCAGATATTGCGTGGTGAG	This study
gKRE5-F	ATGTACTGCGTATGACCTCAGGG	This study
gKRE5-R	TAAAATTGAGGACCGAACTTCATTG	This study
gMNL1-F	TACATAGTTAGAAATCTTCAACGG	This study
gMNL1-R	TTCAAAGAGACTGACGTCGCTGTC	This study
gIRE1-F	TATCGAAGTTGCGAATTCGCACAAC	This study
gIRE1-R	ATATGGTTTTATCAGCGTCCGAG	This study
g*HAC1*-F	AGAGAACGAGCATGTTACCCGACTC	This study
g*HAC1*-R	ATGCTTTCTCCGATTTCGAAGAAG	This study
gHRD1-1-F	TTTACATTGGCAGATGTAGCACGAC	This study
gHRD1-1-R	TCATAATGAGTCCAACCAACGAAGC	This study
gEMP24-F	AAGCCGATCGATCCTAATTACTAGG	This study
gEMP24-R	ACGTTGAAAGTGACATCTTTGGTGG	This study
SEC61-F	ACAACTATCAAAACACAACTAGTGGATCCCCCGGGAAAAATGGTAAGTGTCCAGTTT GATG	This study
SEC61-R	ACATAACTAATTACATGACTCGAGAAGAGATCATTAGTTGATCAACTTTCCTGTC	This study
SBH1-F	ACAACTATCAAAACACAACTAGTGGATCCCCCGGGAAAAATGGTAAGTGTCCAGTTT	This study

Primers	Sequences (5′→3′)	Source
	GATGAGTGCAG	
SBH1-R	TGACATAACTAATTACATGACTCGAGAAGAGATCATTAGTTGATCAACTTTCCTGTCAGCTTAG	This study
SSS1-F	AACTATCAAAACACAACTAGTGGATCCCCCGGGAAAAATGTCCCAAAAAGTCACCGAC	This study
SSS1-R	TGACATAACTAATTACATGACTCGAGAAGAGATCATTAAACAATCAAATATCTGATTG	This study
SEC62-F	AACTATCAAAACACAACTAGTGGATCCCCCGGGAAAAATGGAGCAGGTTCCAGTCGC	This study
SEC62-R	TGACATAACTAATTACATGACTCGAGAAGAGATCATTATTCATCATAAACTTCTTCTATG	This study
SEC63-F	AACTATCAAAACACAACTAGTGGATCCCCCGGGAAAAATGGCACGAGTCGAGTATGATTATG	This study
SEC63-R	ACATAACTAATTACATGACTCGAGAAGAGATCACTATTTATCTTCACCCTCATC	This study
KAR2-F	ACAACTAGTGGATCCCCCGGGAAAAATGCTGTCGTTAAAACCATCTTGGC	This study
KAR2-R	TGACTCGAGCTACAAGTCCTCTTCAGAAATGAGCTTTTGCTCCAACTCATCATGATCATAGTCATAG	This study
LHS1-F	GAACAACTATCAAAACACAACTAGTGGATCCCCCGGGAAAAATGAGAACACAAAAGATAG	This study
LHS1-R	ATAACTAATTACATGACTCGAGTTACAAGTCCTCTTCAGAAATGAGCTTTTGCTCCAACTCATCATGGGATG	This study
SIL1-F	AACTATCAAAACACAACTAGTGGATCCCCCGGGAAAAATGAAAGTGACATTATCTGTG	This study
SIL1-R	TAACTAATTACATGACTCGAGCTACAAGTCCTCTTCAGAAATGAGCTTTTGCTCTAACTCATCGGAGAAACC	This study
PDI1-F	ACAACTAGTGGATCCCCCGGGAAAAATGATGAAGTCGTTACTGCTACTTCTATTAG	This study
PDI1-R	TGACTCGAGTTACAAGTCCTCTTCAGAAATGAGCTTTTGCTCCAATTCCCTTAGTTGGGCATACTCTTC	This study
ERO1-F	TTGAACAACTATCAAAACACAACTAGTGGATCCCCCGGGAAAAATGAGGATAGTAAGGAGC	This study
ERO1-R	ACATATAGAGTAGACTTGTAATGATCTCTTCTCGAGTCATGTAATTAGTTATGTCA	This study
CNE1-F	AACTATCAAAACACAACTAGTGGATCCCCCGGGAAAAATGAAGATCTCTACCATTGC	This study
CNE1-R	ATAACTAATTACATGACTCGAGCTACAAGTCCTCTTCAGAAATGAGCTTTTGCTCGGTTCTCTTTGTAGC	This study
KRE5-F	ACAACTATCAAAACACAACTAGTGGATCCCCCGGGAAAAATGATTAATCATGTCTTAATATTG	This study
KRE5-R	ATAACTAATTACATGACTCGAGTTACAAGTCCTCTTCAGAAATGAGCTTTTGCTCAAGCTCATCATGTTCAG	This study
MNL1-F	AACAACTATCAAAACACAACTAGTGGATCCCCCGGGAAAAATGCTACCAATTAGATG	This study
MNL1-R	ATAACTAATTACATGACTCGAGTTACAAGTCCTCTTCAGAAATGAGCTTTTGCTCGACTACGGATGATACCC	This study
IRE1-F	TTGAACAACTATCAAAACACAACTAGTGGATCCCCCGGGAAAAATGCGATGGTTGATTTGG	This study
IRE1-R	TCTTCGGAGCAATTGGAATGATGATCTCTTCTCGAGTCATGTAATTAGTTATGTCA	This study
HAC1-F	AACTATCAAAACACAACTAGTGGATCCCCCGGGAAAAATGCCCGTAGATTCTTCTC	This study
HAC1-R	TAACTAATTACATGACTCGAGTCACAAGTCCTCTTCAGAAATGAGCTTTTGCTCCCTGATCGCTATGCATG	This study
HRD1-F	TTGAACAACTATCAAAACACAACTAGTGGATCCCCCGGGAAAAATGATATCCATATTTTGG	This study
HRD1-R	CAGCGGCTCAACTCTCAGTAATGATCTCTTCTCGAGTCATGTAATTAGTTATGTCA	This study

Primers	Sequences (5′→3′)	Source
EMP24-F	AACAACTATCAAAACACAACTAGTGGATCCCCCGGGAAAAATGAAGATATTTGTCATCC	This study
EMP24-R	ATAACTAATTACATGACTCGAGTTACAAGTCCTCTTCAGAAATGAGCTTTTGCTCTACAACAGTTTTGACC	This study
SEC13-F	AACTATCAAAACACAACTAGTGGATCCCCCGGGAAAAATGGCAAGTATAGTTTTCCGTACAAGTC	This study
SEC13-R	TGACATAACTAATTACATGACTCGAGAAGAGATCATTATTGATCGACTTCGCCAGCGGAC	This study
SEC23-F	TTGAACAACTATCAAAACACAACTAGTGGATCCCCCGGGAAAAATGGCTGACATCTATGAG	This study
SEC23-R	TGACATAACTAATTACATGACTCGAGAAGAGATCATTAACTACTATTCTGAACCAC	This study
SHL23-F	TTGAACAACTATCAAAACACAACTAGTGGATCCCCCGGGAAAAATGGTAAGAAATCAAAGA	This study
SHL23-R	GTTGTCGTAAAGAGTGTTTAGTGATCTCTTCTCGAGTCATGTAATTAGTTATGTCA	This study
SEC24-F	ACAACTATCAAAACACAACTAGTGGATCCCCCGGGAAAAATGTCTGGAAAGAGAAGAGC	This study
SEC24-R	TGACATAACTAATTACATGACTCGAGAAGAGATCATTAAGAAGACAGTTTTTCACGGATTTG	This study

Table S5. The amino acid and DNA sequence information of PR961 and PR953

	Amino acid sequence	DNA sequence
PR961	QVQLVQSGAEVKKPGASVKLSCKASGYSFTSY<br>WVNWVRQAPGQGLEWIGMIHPSDSETRLNQK<br>FKDRVTITVDKSTSTAYMELSSLRSEDTAVYYCA<br>RADGYEWYFDVWGRGTLVTVSSGGGGSGGGG<br>SGGGGSDIVLTQSPASLAVSPGQRATITCRASESV<br>DSYGNSFMHWYQQKPGQPPKLLIYRASNLESGI<br>PARFSGSGSGTDFTLTINPVEANDVANYYCQQS<br>NEDPWTFGQGTKVEIK	CAAGTTCAATTGGTTCAATCTGGTGCTGAAGTTAAAAAA<br>CCTGGTGCTTCTGTTAAATTGTCTTGTAAAGCTTCTGGTT<br>ATTCTTTTACTTCTTATTGGGTTAATTGGGTTAGACAAGC<br>TCCTGGTCAAGGTTTGGAATGGATTGGTATGATTCATCCA<br>TCTGATTCTGAAACAAGATTGAATCAGAAATTTAAGGAT<br>AGGGTTACTATTACTGTTGATAAATCTACTTCTACTGCTT<br>ATATGGAATTGTCTTCTTTGAGATCTGAAGATACTGCTGT<br>TTATTATTGTGCTAGAGCTGATGGTTATGAATGGTATTTTG<br>ATGTTTGGGGTAGAGGTACTTTGGTTACTGTTTCTTCTGG<br>TGGTGGTGGTTCTGGAGGTGGAGGTTCTGGTGGAGGTG<br>GATCTGATATTGTTTTGACTCAATCTCCTGCTTCTTTGGCT<br>GTTTCTCCTGGTCAAAGAGCTACTATTACTTGTAGAGCTT<br>CTGAATCTGTTGATTCTTATGGTAATTCTTTTATGCATTGG<br>TATCAACAAAAACCTGGTCAACCACCAAAATTGTTGATT<br>TATAGAGCTTCTAATTTGGAATCTGGTATTCCTGCTAGAT<br>TTTCTGGTTCTGGTTCTGGTACTGATTTTACTTTGACTATT<br>AATCCTGTTGAAGCTAATGATGTTGCTAATTATTATTGTC<br>AACAATCTAATGAAGATCCATGGACTTTTGGTCAAGGTA<br>CTAAAGTTGAAATTAAA
PR953	EVQLQQSGPELVKPGASVKISCKTSGYTFTEYTM<br>HWVKQSHGKSLEWIGGINPNNGDNTYNQKLK<br>GKATLTVHKSSSTAYMELRSLTSEDSAVYYCARD<br>GYPYYYALDYWGQGTSVTVSSGGGGSGGGGSG<br>GGGSDIVMTQSQKFMSTSVGDRVSVTCKASQN<br>VGTNVAWYQQKPGQSPKPLIYSASSRYSGVPDR<br>FTGSGSGTDFTLTISNVQSEDLAEYFCQQYNNY<br>PWTFGGGTKLEIK	GAAGTTCAATTGCAACAATCTGGTCCTGAATTGGTTAAA<br>CCTGGTGCTTCTGTTAAAATTTCTTGTAAAACTTCTGGTT<br>ATACTTTTACTGAATATACTATGCATTGGGTTAAACAATC<br>TCATGGTAAATCTTTGGAATGGATCGGTGGAATTAATCC<br>AAACAACGGAGACAATACTTATAATCAAAAATTGAAAG<br>GTAAAGCTACTTTGACTGTTCATAAATCTTCATCTACTGC<br>TTATATGGAATTGAGATCTTTGACTTCTGAAGATTCTGCT<br>GTTTATTATTGTGCTAGAGATGGTTATCCATATTACTATGC

TTTGGATTATTGGGGTCAAGGTACTTCTGTTACTGTTTCTT
CTGGAGGTGGAGGATCTGGTGGTGGAGGTTCTGGTGGA
GGTGGATCTGACATCGTTATGACTCAATCTCAAAAATTT
ATGTCTACTTCTGTTGGTGATAGAGTTTCTGTTACTTGTA
AAGCTTCTCAAAATGTTGGTACTAATGTTGCTTGGTATCA
ACAAAAACCTGGTCAATCTCCAAAACCATTGATTTATTC
TGCTTCTTCTAGATATTCTGGTGTTCCTGATAGATTTACTG
GTTCTGGTTCTGGTACTGATTTCACTTTAACTATTTCTAAC
GTTCAATCTGAAGATTTGGCTGAGTATTTTTGTCAACAAT
ATAATAATTATCCATGGACTTTTGGTGGAGGTACTAAATT
GGAAATTAAA

Table S6. The gRNA target sequences of genes

Gene name	gRNA1	gRNA2	gRNA3	Source
SIL1	GTTAGAATCGTTTGTTCGGA	GTTAGCTATTGCCTCCCAAT	ATTGGTTAGAATCGTTTGTT	This study
PDI1	CAACTGGAATATTAAAACTG	CTCTCACACTAGCACAAGCA	ACTAGCACAAGCAAGTGATC	This study
CNE1	GGGTCTGAACTCAGCGGGTT	GAACTCAGCGGGTTCGGATT		This study
KRE5	ATATCAGTTTGAAGGCCAAT	GTTTGTTGATATCAGTTTGA	TCCTTTCCCACTGTTACTAC	This study
MNL1	ATTAGATGTGGGTATGTCTC	TGTCTCTAGGTACTCTGATG	AGCTGCTCAAGAATGAGACG	This study
IRE1	GCAGAGCTTCCTTGTTATAC	ATCTGCGTTTACTCATAATG		This study
HAC1	AGCAAAGACGGAAGAAGAAA	TATCCTACGTAATAGGAGAG		This study
HRD1	CAGGATCCGAAGCCAAAATA	TGATATCCATATTTTGGCTT		This study
EMP24	GAATTGCTTCCTCTGGGAAC	CTGGGAACTGGTCTTATTGT	CTACCAAGAATTGCTTCCTC	This study

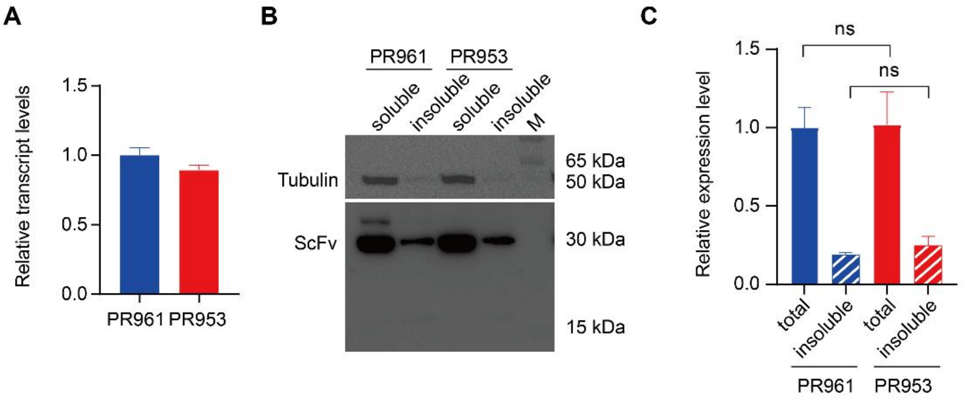

Figure S1. Transcript levels and intracellular protein levels of PR961 and PR953 in their respective recombinant strains (V_H-linker-V_L, 2-copies). (A) Relative mRNA expression of PR961 and PR953 at 24 hours is shown relative to 0 hours. The values were normalized against the PR961. (B) Western Blot (WB) images of intracellular PR961 and PR953 expression. (C) Quantification of intracellular PR961 and PR953 expression using the ImageJ software. The values were normalized to the total expression level (sum of insoluble and soluble) of PR961. ns, no statistical significance.

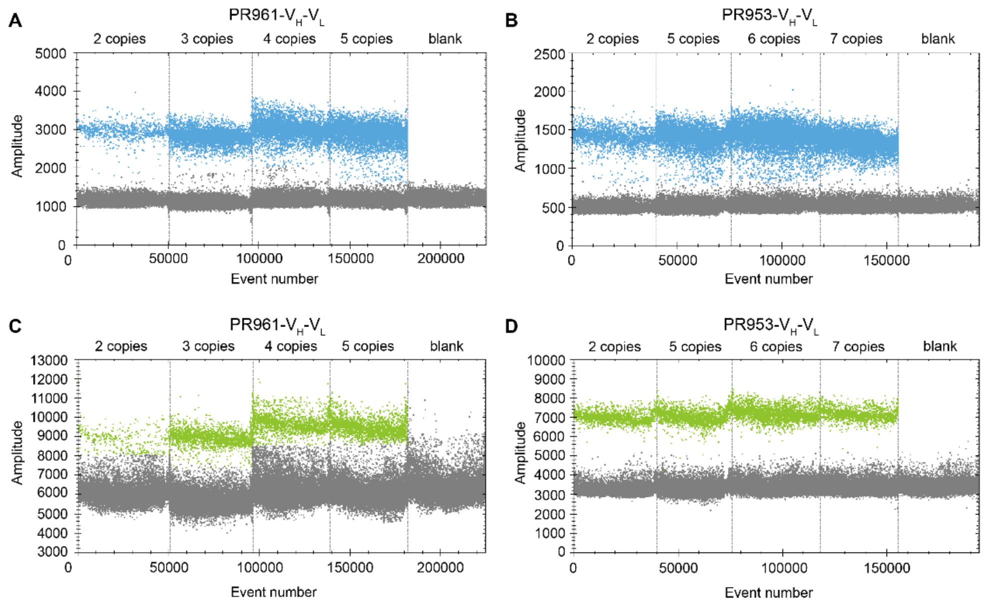

Figure S2. Quantitative droplet-digital PCR (ddPCR) copy number assays for PR961 and PR953 (VH-linker-VL). The ddPCR results for the (A) *PR961* and (B) *PR953* with a probe labeled by fluorescein phosphoramidite (FAM) in assays using scFv-specific probe. (C) ddPCR results for the housekeeping gene actin using a phosphoramidite (VIC) -labeled housekeeping gene actin-specific probe in the same cases as in (A). (D) ddPCR results for the housekeeping gene actin using a phosphoramidite (VIC) -labeled housekeeping gene actin-specific probe in the same cases as in (B).

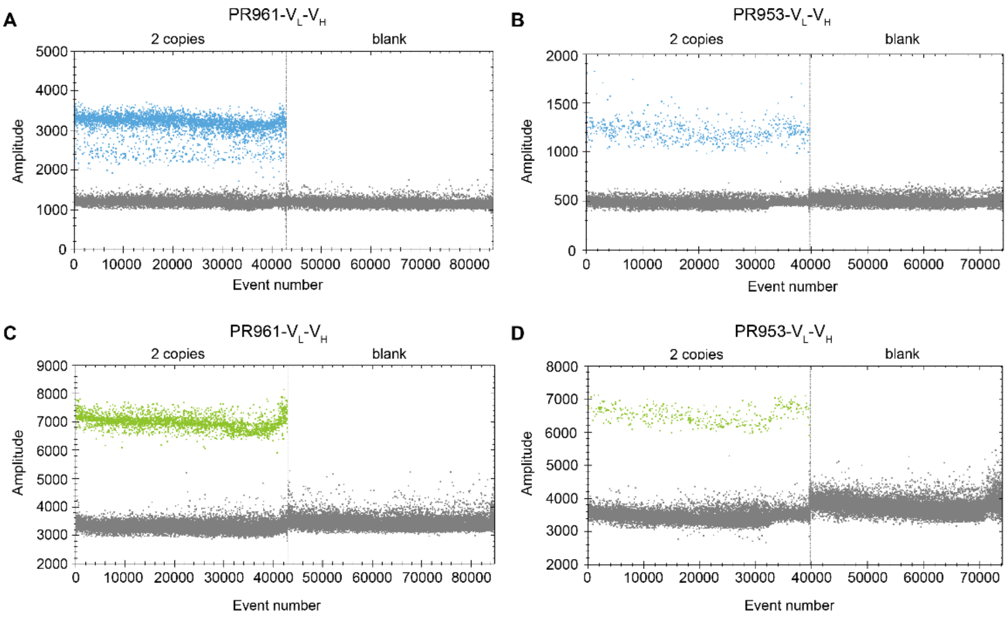

Figure S3. Quantitative ddPCR copy number assay for PR961 and PR953 (VL-linker-VH). The ddPCR results for the (A) *PR961* and (B) *PR953* with a probe labeled by fluorescein phosphoramidite (FAM) in assays using scFv-specific probe. (C) ddPCR results for the housekeeping gene actin using a phosphoramidite (VIC) -labeled housekeeping gene actin-specific probe in the same cases as in (A). (D) ddPCR results for the housekeeping gene actin using a phosphoramidite (VIC) -labeled housekeeping gene actin-specific probe in the same cases as in (B).

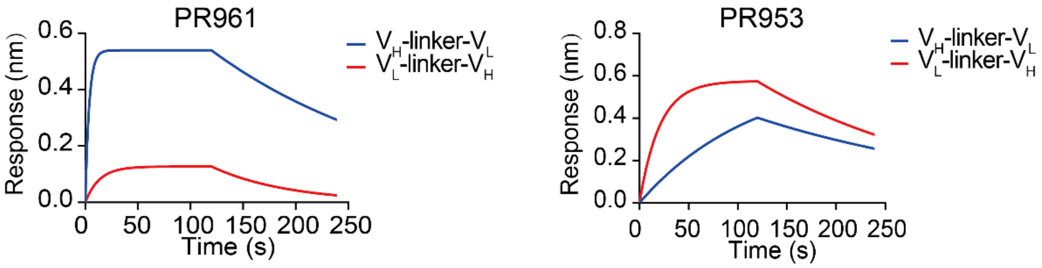

Figure S4. Binding kinetics of PR961 and PR953 in VH-linker-VL and VL-linker-VH orientations against SARS-CoV-2 RBD were measured by biolayer interferometry. Data are shown for single experiments representative of at least three independent experiments.

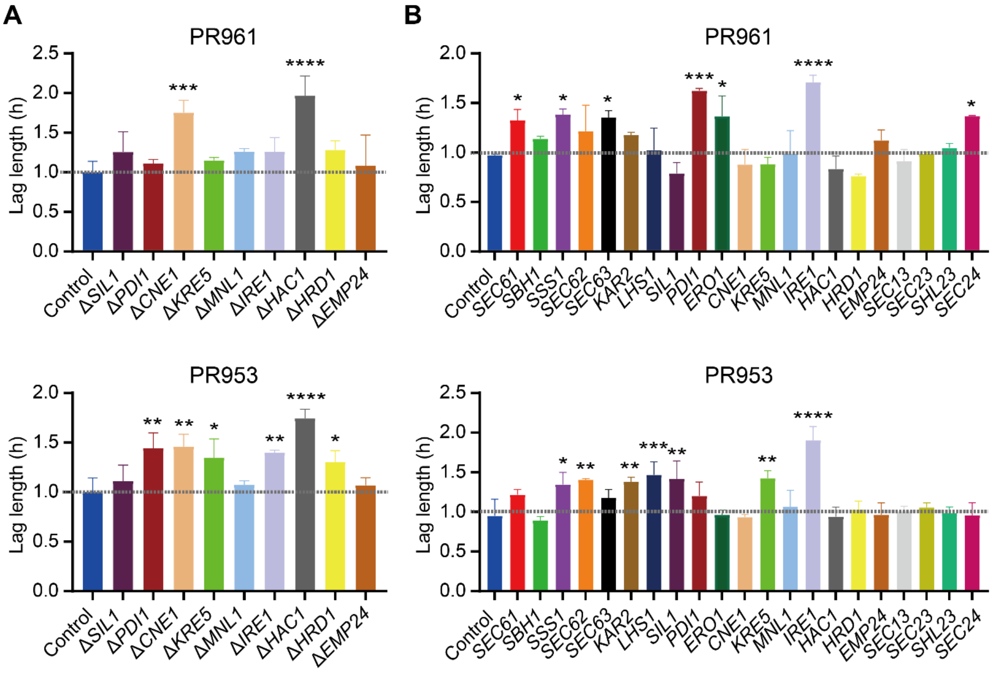

Figure S5. Effects on lag-phase lengths of PR961 and PR953 strains by (A) disrupting or (B) overexpressing ER-related genes. The statistical significance between groups was calculated by one-way ANOVA. Statistical significance levels are denoted as follows: $p < 0.05$ (*), $p < 0.01$ (**), $p < 0.001$ (***), and $p < 0.0001$ (****).

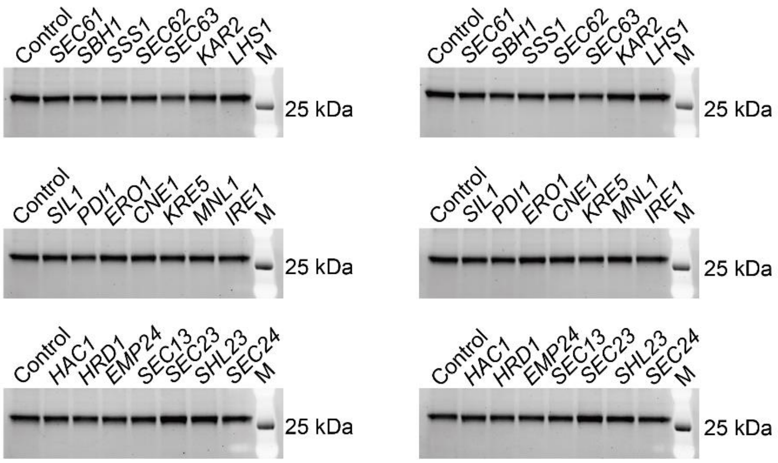

Figure S6. SDS-PAGE analysis of PR961 secretion levels in three biological replicates of gene overexpression. The gels were stained using the SYPRO dye method.

Reference

1. Besleaga, M.; Vignolle, G.A.; Kopp, J.; Spadiut, O.; Mach, R.L.; Mach-Aigner, A.R.; Zimmermann, C. Evaluation of reference genes for transcript analyses in Komagataella phaffii (Pichia pastoris). *Fungal biol. biotechnol*. **2023**, 10, 7. https://doi.org/10.1186/s40694-023-00154-1.
